# Quorum Quenching of *P. aeruginosa* by *Portulaca oleracea* Methanolic Extract and Its Phytochemical Profile

**DOI:** 10.3390/pathogens14020163

**Published:** 2025-02-07

**Authors:** Hala I. Al-Daghistani, Sina M. Matalqah, Khalid A. Shadid, Lubna F. Abu-Niaaj, Sima Zein, Raeda M. Abo-Ali

**Affiliations:** 1 Department of Medical Laboratory Sciences, Faculty of Allied Medical Sciences, Al-Ahliyya Amman University, Amman 19328, Jordan; h.aldaghistani@ammanu.edu.jo; 2 Pharmacological and Diagnostic Research Centre, Faculty of Pharmacy, Al-Ahliyya Amman University, Amman 19328, Jordan; s.matalqah@ammanu.edu.jo (S.M.M.); kshadid@ammanu.edu.jo (K.A.S.); 3 Department of Agricultural and Life Sciences, College of Engineering, Science, Technology and Agriculture, Central State University, Wilberforce, OH 45384, USA; 4 Department of Pharmaceutical Biotechnology, Faculty of Allied Medical Sciences, Al-Ahliyya Amman University, Amman 19328, Jordan; s.zein@ammanu.edu.jo; 5 Faculty of Nursing, Al-Balqa Applied University, Amman 19117, Jordan; raeda.aboali@bau.edu.jo

**Keywords:** *Portulaca oleracea*, *Chromobacterium violaceum*, *Pseudomonas aeruginosa*, quorum sensing, quorum quenching, virulence factors, biofilm, pyocyanin, natural products

## Abstract

Quorum sensing (QS) is a molecular communication mechanism among bacterial cells. It is critical in regulating virulence factors, motility, antibiotic resistance, and biofilm formation. *Pseudomonas aeruginosa* is a Gram-negative opportunistic pathogen linked to healthcare-associated infections, food poisoning, and biofilm formation. Treating infections caused by pathogenic bacteria has become a challenge due to the development of multi-antibiotic resistance upon continuous exposure of bacteria to antibiotics. An alternative strategy to conventional antimicrobials to decrease the bacterial pathogenicity is QS inhibition, also known as quorum quenching. Using plant-derived compounds is an environmentally friendly strategy to block the bacterial QS and inhibit bacterial growth. *Portulaca oleracea* is a popular plant in different countries and is also used in traditional medicine. It is widely consumed raw in salads and as garnishes, though it can be cooked as a vegetarian dish. This study evaluates the antimicrobial activity of the methanolic extract of *P. oleracea* and its effectiveness in blocking or attenuating the QS of *P. aeruginosa*. The agar well diffusion method used for screening the antibacterial activity showed a significant growth inhibition of *P. aeruginosa* by the extract at 500 mg/mL with a minimum inhibitory concentration of 31.25 mg/mL. A bioindicator bacterium, *Chromobacterium violaceum* CV026, was used to determine the effect of the methanolic extract on the QS of *P. aeruginosa.* The results indicated a significant reduction in biofilm formation, pyocyanin production, and LasA staphylolytic activity. The phytochemical analysis by Gas Chromatography–Mass Spectrometry showed that the methanolic extract contained several phenols, alkaloids, esters, and other compounds previously reported to have antibacterial and antioxidant effects. These findings highlight the effectiveness of *P. oleracea* methanolic extract in attenuating the QS and virulence factors of *P. aeruginosa.* This study suggests that *P. oleracea* is an important source of natural antimicrobials and its use would be beneficial in food and pharmaceutical applications.

## 1. Introduction

Quorum sensing (QS) is a molecular communication mechanism among bacterial cells. It is a cell density-based intercellular communication system that regulates the expression of bacterial virulence factors, motility, and biofilm formation [1]. An innovative strategy to decrease the level of bacterial pathogenicity is to inhibit QS, which is known as quorum quenching [2]. *Pseudomonas aeruginosa* is an opportunistic pathogen linked to conjunctivitis, otitis media, and other healthcare-acquired infections such as ventilator-associated pneumonia, post-surgical infection, and burn-wound contamination [3,4]. Treatment can be challenging due to *Pseudomonas’* adaptation to environmental changes, which leads to the development of multi-antibiotic resistance, biofilm formation, and modification of the gene expression of several virulence factors regulated by QS [5]. The virulence factors are mainly exotoxin A, glycolipid biosurfactant (rhamnolipids), siderophores, pigments, and some critical enzymes such as elastase, alkaline protease, and lipases [6,7]. Studies have indicated that the QS regulatory network reacts to environmental stress in bacterial populations. It relies on the secretion and perception of small autoinducer signaling molecules known as acyl homoserine lactones (AHLs), which stimulate bacterial cells to sense and regulate the expression of virulence factors [8,9]. The QS network of *P. aeruginosa* is a multi-layered hierarchy consisting of three main interconnected signaling mechanisms: lasA, rhl, and pqs [1]. The coordinated expression of these genes is triggered when the level of AHLs is above a threshold concentration, activating specific transcription factors [10]. Quorum quenching and interference with the QS system are novel eco-friendly strategies to prevent antibiotic resistance by preventing microbial growth without causing bacterial stress from antibiotic exposure.

Plants and herbs are rich in secondary metabolites, which possess a wide range of physiological activities, including antimicrobial, anticancer, antioxidant, and antispasmodic effects [11,12,13,14,15,16]. These bioactivities are due to different categories of natural compounds, such as polyphenols, flavones, quinones, terpenoids, alkaloids, and volatile oils [13,14,15,16,17]. Many plant extracts have been reported to inhibit microbial growth, interfere with quorum sensing, and prevent biofilm formation in Gram-negative bacteria, including *P. aeruginosa* [11,18,19,20]. For example, acyl homoserine lactones (AHLs) interfere with the autoinducer receptors in bacteria and disrupt cell signaling [21,22]. Studies have suggested that the bioactivity of some natural compounds is due to their structural similarity to cellular enzymes or their substrates, making them function as analogs to such molecules [15].

*Portulaca oleracea* L. (Purslane) is eaten fresh or cooked for its nutritional value, and it is commonly used to treat multiple microbial infections. Chemical studies have indicated that *Purslane* contains polyphenols, alkaloids, α-tocopherol, ascorbic acid, omega-3 fatty acids, β-carotene, and glutathione, in addition to a high content of minerals such as calcium, iron, zinc, magnesium, and potassium [13,23,24,25,26,27]. Several natural products have shown anti-inflammatory, antioxidant, anticancer, hypocholesterolemia, and bronchodilator properties [24,28,29,30,31,32,33]. However, to the authors’ knowledge, no study has yet identified the mechanism for such activities. This study evaluates the effect of the methanolic extract of *P. oleracea* on QS by *P. aeruginosa* cells and its influence on the biofilm formation and expression of other virulence factors of this bacterium.

## 2. Materials and Methods

All chemicals were purchased from Thermo Fisher Scientific (Waltham, MA, USA), unless mentioned otherwise.

### 2.1. Plant Collection and Preparation of Methanolic Extract

**Figure 1:**
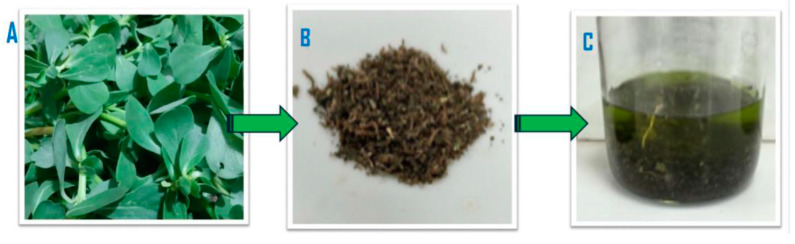
*Portulaca oleracea* (Purslane) (**A**); air-dried plant (**B**); leaves soaked in methanol (**C**).

*Portulaca oleracea* was purchased from local stores in Amman, Jordan. The plant was authenticated by the National Agricultural Research Center in Jordan. The leaves were collected and thoroughly washed with distilled water to remove impurities. They were then air-dried at room temperature, ground in an electric grinder, and stored in an airtight container until needed. A methanolic extract was prepared by soaking 500 g of ground material in 1 L of methanol at room temperature with continuous agitation for three days (Figure 1). The mixture was then filtered using Whatman No. 1 filter paper to remove larger particles, and the filtrate was concentrated through vacuum evaporation at 45 °C to yield a dry material referred to as the “crude”. For antimicrobial activity screening, a stock solution of the crude was prepared at a concentration of 500 mg/mL by dissolving the desired amount of the dry material in a physiological buffer solution (PBS). The serial solutions used in this study were prepared using this stock solution.

### 2.2. Bacterial Samples and Growth Conditions

The antibacterial activity of *P. oleracea* and its antibiofilm effectiveness were evaluated against *Pseudomonas aeruginosa* (ATCC27853) (American Type Culture Collection, Manassas, VA, USA). The effect of the extract on QS was assessed on a clinical isolate of *P. aeruginosa* collected from sputum for another study, and the concentration of its produced pyocyanin was 36.448 (μg/mL) (IRB # BAU/24/11/2022-2023). *Staphylococcus aureus* (ATCC 25923) was used as a reference organism in the LasA assay. Upon use, a bacterial inoculum in LB broth was incubated overnight at 30 °C [34]. The biosensor strain used was *Chromobacterium violaceum* CV026 (Carolina Biological Supplies, Burlington, NC, USA), a Tn5 mutant strain derived from wild-type *C. violaceum* (CV31532). This mutant bacterium cannot produce N-acyl homoserine lactones (AHLs), a class of small signaling molecules involved in bacterial quorum sensing, however, it remains responsive to exogenous AHLs, such as N-hexanoyl-L-homoserine lactone (C6-AHL) and N-butanoyl-L-homoserine lactone (C4-HSL) [22]. It produces a purple-violet pigment called violacein, which indicates cellular communications [35]. AHLs are signaling molecules involved in bacterial quorum sensing by regulating gene expression in Gram-negative bacteria. For use, *C. violaceum* was inoculated in LB containing 1% tryptone, 0.5% yeast extract, and 1% NaCl and incubated at 30 °C for 48 h [36], and the optical density at OD_600_ was adjusted to 0.1–0.2 (equivalent to 0.5 McFarland, which represents 1.5 × 10^8^ CFU/mL).

### 2.3. Effect of Methanolic Extract on P. aeruginosa

#### 2.3.1. Screening of Antibacterial Activity

The inhibitory effect of the *P. oleracea* methanolic extract against *P. aeruginosa* was assessed according to the guidelines of the Clinical and Laboratory Standards Institute [37].

A solution of the crude extract at a concentration of 500 mg/mL was used for the evaluation, and the effect was compared to standard antibiotics. Mueller–Hinton (MH) agar plates were used, and 8 mm diameter wells were made in the agar plates using a sterile borer. In the MH broth, a bacterial inoculum was grown overnight at 37 °C, and the OD_600_ was standardized to 0.1. A sterile cotton applicator was immersed in the standardized culture and swabbed uniformly on the MH agar plates. Then, 125 μL of the stock solution of the plant extract was placed in a well, while the PBS was placed in a control well because it was the solution used to dissolve the crude. Plates were incubated at 37 °C for 24 h, and the diameters of inhibition zones were measured in millimeters (mm). The inhibition was reported as an average reading of three replicates. The inhibition of bacterial growth was compared to that caused by standard antibiotics. For the comparison study, disks of the selected antibiotics were distributed on the surfaces of agar plates swabbed with *P. aeruginosa* and incubated overnight at 37 °C before measuring the diameters of inhibition zones in mm. The antibiotic disks contained penicillin G (10 μg), clarithromycin (15 μg), ciprofloxacin (5 μg), lincomycin (2 μg), cephalexin (30 μg), amoxicillin (25 μg), tetracycline (10 μg), azithromycin (15 μg), and clindamycin (2 μg).

#### 2.3.2. Determination of Minimum Inhibitory Concentration (MIC)

The MIC of the *P. oleracea* crude (dry) of the methanolic extract was determined using a series of six (6) two-fold dilutions (250, 125, 62.5, 31.25, 15.62, and 7.81 mg/mL) prepared from the 500 mg/mL stock; then, they were filtered through 0.45 µm filters. In the MH agar, wells of 8 mm were made; a well for each dilution plus a well for the control. A standardized bacterial inoculum was spread on the agar surface, and 125 μL of each dilution or the control solution (PBS) was placed in the wells. The agar plates were incubated at 37 °C overnight before measuring the inhibition zones in mm.

### 2.4. Inhibitory Effects of P. oleracea on the Virulence Factors of P. aeruginosa

For the assays below, the sub-MIC was used, and this concentration is defined as the extract’s concentration before the MIC that allowed for bacterial growth. The growth of *P. aeruginosa* was confirmed by incubating a bacterial inoculum in MH broth to grow overnight at 37 °C, while the absorbance at OD_600_ was measured every 2 h.

#### 2.4.1. Evaluation of *P. oleracea* Effect on Quorum Sensing

To determine the potential of the methanolic extract as an inhibitor for quorum sensing in *P. aeruginosa*, the mutant bacterium *C. violaceum* CV026 was used as a biosensor for cell density, indicating QS [34]. This bacterium secretes a purple pigment called violacein, but only when exposed to exogenous 3-oxo-C6-HSL and short-chain AHLs [38]. Thus, a reduction in violacein production indicates an interruption of quorum sensing or complete quorum quenching. The assay was performed by topping the MH agar plates with a thin-layer of agar containing *C. violaceum* prepared by adding 10 mL of a culture of this bacterium to 200 mL of MH semisolid agar supplemented with 2 mL of 10 μM synthetic AHL called Acetyl-L-homoserine lactone (CAS 51524-71-1) from Santa Cruz Biotechnology, Inc. (Dallas, Texas, U.S.A.). The agar mixture was poured onto pre-warmed MH agar plates and left to solidify at room temperature before making 8 mm wells in the agar. The wells contained either 125 μL of sub-MIC of plant extract or a control. In addition to PBS as a control, Furanone or methanol were used as positive and negative controls, respectively. Inhibitory zones smaller than 10 mm indicated moderate activity, while zones bigger than 10 mm indicated potent activity [39]. The assay was also performed by placing 125 μL tetracycline (10 µg) in a well as a control to evaluate the antibiotic effect on QS activity.

#### 2.4.2. Inhibition of Biofilm Formation

The effectiveness of the plant extract as an inhibitor for biofilm formation was evaluated according to Adeyemo et al., with modifications [40]. In a microtiter plate, a culture of *P. aeruginosa* was grown in Tryptic Soy broth at 37 °C overnight. The plate was centrifuged at 4500 rpm for 15 min and washed twice with PBS; then, the bacterial cells were resuspended in LB broth to obtain an OD_600_ of 0.1–0.2. To each well in a 96-microtiter plate, 180 μL of sterile LB broth was added; then, 150 μL of standardized bacterial culture (approximately 1.5 × 10^8^ CFU/mL) was added and mixed well. A 50 µL sub-MIC solution was added making the total volume 380 μL per well, while the control well had 50 µL of PBS (the solution used to prepare the sub-MIC). Biofilm formation was initiated by incubating the 96-well plate at 37 °C overnight, and then the cell density was determined quantitatively using a crystal violet stain. The plate was washed gently three times with sterile distilled water and dried. Following this, 200 μL of 0.2% crystal violet was added to each well, and the microtiter plate was incubated at room temperature for 15 min. The plate was washed to remove the excess stain, and 100 μL of 95% ethanol was added and mixed before reading the absorbance at 570 nm. The experiment was performed in triplicate, and data were presented as averages. The percentage of biofilm inhibition was calculated using the following equation:

Inhibition of Biofilm Formation (%) = [(OD_570_ of Control − OD_570_ of Treated wells)/OD_570_ of Control] × 100

#### 2.4.3. Inhibition of Staphylolytic LasA

The assessment of the effect of the plant extract on the activity of LasA of *P. aeruginosa* was carried out according to the method of Alasil et al. with modifications [34]. LasA is an extracellular protease secreted by *P. aeruginosa* that has multiple roles in bacterial virulence, including the lysis of *Staphylococcus aureus*. It is measured by the ability of a *P. aeruginosa* supernatant to lyse boiled cells of *S. aureus.*

Briefly, an overnight culture of *P*. *aeruginosa* was grown in LB medium at 37 °C in a shaker. The culture was divided into 10 mL aliquots, to which 1 mL of fresh LB medium containing a sub-MIC dilution of *Portulaca* was added to a final concentration of 1 mg/mL. After approximately 12 h, when the bacterial culture was expected to be in a late stationary phase, the culture was centrifuged at 10,000× *g* for 10 min, and the supernatant was used in the assay. The control was the cell supernatant without the addition of the plant extract. An overnight culture of *S. aureus* was boiled for 10 min and centrifuged for 10 min at 13,000 rpm, and the pellet was resuspended in 10 mM of sodium–phosphate buffer (Na_2_PO_4_ with pH 4.5). The culture was read at OD_600_ and standardized to 0.1 (0.5 McFarland). A 100 µL aliquot of the *P. aeruginosa* supernatant with or without the plant crude extract was added to 900 µL of the boiled *S. aureus* suspension. The positive control was 2(5H)-Furanone, and LB broth was used as a negative control. The OD_600_ was recorded every 10 min for 1 h, and the staphylolytic LasA activity was expressed as the OD_600_ change per hour per μg of protein.

#### 2.4.4. Phytochemical Analysis by Gas Chromatography-Mass Spectrometry

The analysis of the chemical composition of the *P. oleracea* methanolic extract was carried out by Gas Chromatography–Mass Spectrometry (GC-MS). The system consisted of an HP 5890 series II plus GC and 5972 quadrupole mass selective detector (MSD) coupled to a Vectra XM2 4/100i computer workstation. A sample of the crude extract was reconstituted in 1 mL of dichloromethane ≥ 99.8% (*v*/*v*) (Aldrich Chemical, St Louis, MO, USA). The sample passed through glass wool to remove the remaining solid materials, and 2.0 μL was transferred into an autosampler glass vials with Teflon caps for analysis. The column used was a DB-5 ms column, with dimensions of 30 cm × 0.25 μm × 0.25 mm. The instrument settings were as follows: the analysis time was 50 min, the injector temperature was set to 250 °C with the injection mode set to Spitless, and the temperatures of the ion source and interface were set to 200 °C and 280 °C, respectively. The identification of components via the mentioned software was carried out to compare the spectra of unknown compounds to standard database spectra from a library. The relative amount of each compound was expressed as a percentage of the total peak area in the chromatogram, based on the retention time.

### 2.5. Statistical Analysis

All the bioassays were performed in triplicate, and the data were presented as mean values with or without the standard deviation (SD). Data analysis was performed with SPSS software version 19.0 (Chicago, IL, USA). The statistical difference between different test conditions was determined using Student’s *t*-test. The difference was considered significant when *p* < 0.05.

## 3. Results

### 3.1. Plant Crude Extract

The evaporation of the 500 g plant filtrate in 1 L of methanol resulted in approximately 64 g of crude extract. The yield calculated using the formula below was 12.77%

$$Yield\left(\%\right)=\frac{Weight\;of\;the\;crude\;after\;evaloration}{Weight\;of\;initial\;raw\;material}\times 100\%$$

### 3.2. Effect on Bacterial Growth

**Figure 2:**
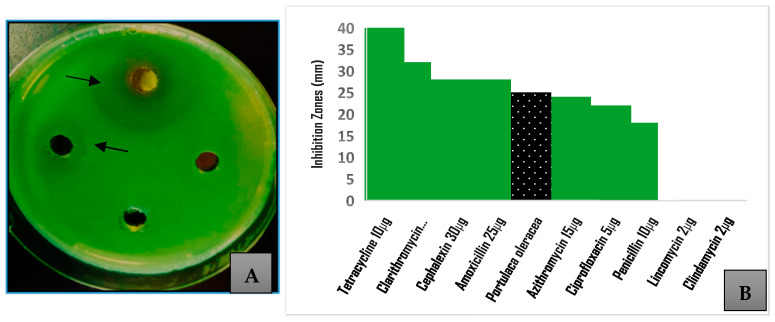
Antibacterial activity screening of *Portulaca oleracea* methanolic extract; (**A**) inhibition zones exhibited by different concentrations of the crude extract against *Pseudomonas aeruginosa* (arrows); (**B**) comparison of inhibition caused by 500 mg/mL of crude extract to that exhibited by selected antibiotics.

The methanolic extract at a concentration of 500 mg/mL was evaluated for its antibacterial activity against *P. aeruginosa.* The agar well diffusion method showed a growth inhibition of 25 ± 2.1 mm. Figure 2 shows that the inhibition zone was higher than that caused by several antibiotics used to treat *P. aeruginosa* infections (*p* = 0.001), including penicillin, ciprofloxacin, azithromycin, lincomycin, and clindamycin. The inhibition was 62% of that caused by tetracycline, which exhibited the highest inhibition (40 mm) of bacterial growth among the tested antibiotics. The MIC was determined to be 31.25 mg/mL, making the sub-MIC 62.5 mg/mL. This sub-MIC solution was used in the assays performed to evaluate the effect of the *Portulaca* methanolic extract on the expression of the virulence factors.

### 3.3. Effects of P. oleracea on the Virulence Factors of P. aeruginosa

#### 3.3.1. Effect on Quorum Sensing

**Figure 3:**
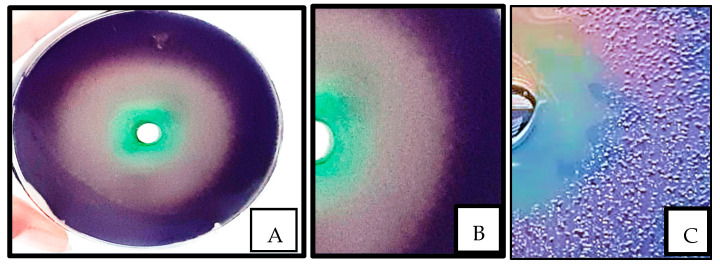
Evaluation of quorum sensing; (**A**) whole plate revealing anti-QS activity of *Portulaca oleracea* extract using *Chromobacterium violaceum*; (**B**) a confluent layer of bacteria, which lost their ability to produce violacein upon exposure to *P. oleracea*; (**C**) the growth inhibition zone surrounding the well having tetracycline (10 μL).

A clinical isolate of *P. aeruginosa* was used to determine the effect of the extract on QS. Figure 3 shows that the sub-MIC exhibited significant inhibition of violacein, with an inhibition zone of 68 mm, as indicated by the appearance of a colorless murky halo zone around the well. When the screening was performed using tetracycline, a clear inhibition zone appeared around the well, indicating bacteriostatic activity, while the region surrounding the well containing the sub-MIC extract showed normal bacterial growth.

#### 3.3.2. Biofilm Formation

The assessment of the attachment phase of the biofilm in response to the plant extract was carried out using the sub-MIC. The biofilm formation was significantly reduced (67.08%) by the tested concentration of the methanolic extract (*p* < 0.05). This reduction was indicated by the decreased absorbance at OD_570_ nm for the plant-treated well (0.186 ± 0.040) compared to that measured for the control (0.565 ± 0.012).

#### 3.3.3. Staphylolytic LasA Inhibition Assay

**Figure 4:**
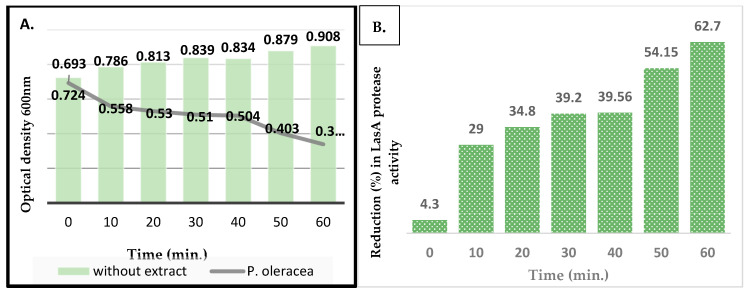
The activity of LasA protease of *P. aeruginosa* with and without *P. oleracea* methanolic extract; (**A**) The OD_600_ for the activity of LasA protease at different time intervals; (**B**) the percentage of reduction in activity of LasA protease with time in the presence of the extract.

Figure 4 shows the reduction in activity for LasA protease with time for the bacterial cultures inoculated with the sub-MIC of the plant extract. The OD_600_ readings indicated a significant time-dependent decrease in the activity of LasA protease upon treating cells with the plant extract. The OD_600_ for the negative control (LB) was 0.635, compared to 0.207 for the positive control, which was 2(5H)-Furanone.

### 3.4. Phytochemical Analysis by Gas Chromatography-Mass Spectrometry (GC_MS)

**Figure 5:**
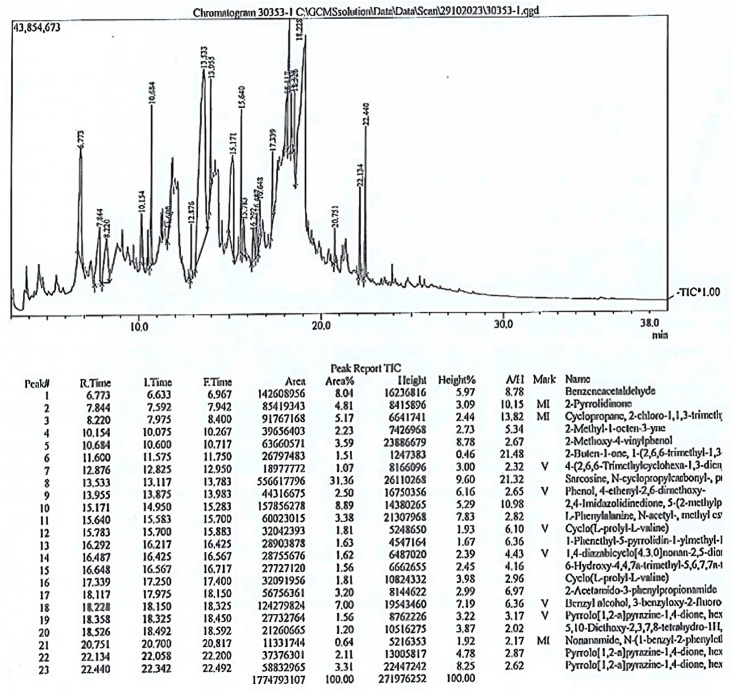
GC-MS chromatogram of *Portulaca aleracea* methanolic extract.

The GC-MS analysis of the phytochemical composition of the methanol extract of *P. oleracea* indicated the presence of twenty-two compounds (Table 1). The mass spectrum of the compounds is presented in Figure 5.

## 4. Discussion

*P. aeruginosa* is a common leading cause of infections in healthcare settings and the second leading cause of pneumonia in patients supported by ventilators in the United States [60]. The pathogenicity of this bacterium is largely attributed to several virulence factors that enhance the colonization and invasion of the host tissues and thus its survival. *P. aeruginosa* is a biofilm-forming bacterium that utilizes QS signals to establish colonies and evade the host immune system [61]. Pathogens, such as *P. aeruginosa,* capable of biofilm formation pose a significant challenge in the medical field due to the exhibition of high resistance to both antibiotics and host immune responses [62,63,64]. It was reported that disrupting biofilm formation and reducing virulence factors regulated by QS is a great approach to controlling microbial infection. Therefore, attenuating or blocking the expression of virulence factors is an innovative strategy to replace traditional antibiotics to decrease microbial pathogenicity. The quorum sensing system controls these factors as it represents cellular communication at the molecular level. This study suggests that the methanolic extract of *Portulaca oleracea* caused quorum quenching, as indicated by the decreased expression of the virulence factors, the production of pyocyanin, the LasA activity, and biofilm formation. Studies have reported that extracts of several plants contain inhibitors for QS [65,66], while others have been recognized for their inhibitory effect on bacterial growth and biofilm formation [11,18,67,68]. Such bioactivities have been attributed to natural products such as polyphenols, tannins, and alkaloids [14,15,16,17,18]. It has been reported that some phenols compete with AHLs for QS receptor binding, causing the inhibition of the QS system [69,70,71], while alkaloids have been reported as homologs to AHLs [72].

*P. oleracea* is recognized by the World Health Organization as a popular medicinal plant used for treating microbial infections [73,74]. This plant is rich in nutritional value with no reported side effects on health [75]. The methanolic extract of *Portulaca* showed an abundance of polyphenols, which are antioxidants and exhibit antimicrobial properties [76]. The antimicrobial activity of different extracts of *Portulaca* was reported in comparison to *Candida albicans* and several Gram-positive and Gram-negative bacteria, including *P. aeruginosa* [28,30,33,77].

QS-inhibitory molecules are interesting when addressing *P. aeruginosa* infections due to their ability to mitigate inflammation-induced damage and counteract the growing issue of antimicrobial resistance [78]. Biological compounds typically disrupt the bacterial AHL-QS system in three ways: inhibiting the synthesis of signaling molecules through the LuxI-encoded AHL synthase, degrading or modifying the signaling molecules, or targeting the LuxR signal receptor [79]. Our study reports a significant reduction in biofilm formation due to *P. aeruginosa*, with a decrease of 67.08% when cultured in the presence of the *P. oleracea* extract. The inhibitory effect of this methanolic extract may be due to small molecules being extracted by methanol that possibly interfere with the AHL signaling molecules, either through their degradation or modification by specific proteins or by antagonizing their activity.

The phytochemical analysis by GC-MS revealed a diverse array of secondary metabolites in the *P. oleracea* methanolic extract, encompassing phenols, ketones, fatty acids, alkaloids, and inorganic and organic compounds (Table 1). The analysis showed the richness of the plant in several compounds previously reported for their antibacterial and antioxidant activities. Among these bioactive compounds was pyrrolo [1,2-a] pyrazine-1,4-dione, hexahydro-3-(2-methylpropyl) (C_14_H_16_N_2_O_2_), which has previously been reported for its significant anti-quorum sensing activity against *P. aeruginosa.* Its effective inhibition of biofilm formation contributed to modifications in the architecture of the biofilm, thus hindering bacterial adherence and subsequent biofilm development while preserving cell viability within the biofilm matrix. Furthermore, this compound has been reported to decrease the motility of *P. aeruginosa* cells and reduce the expression of virulence factors such as pyocyanin, rhamnolipid, and other enzymes including elastase and proteases [80]. Another compound that might contribute to the inhibitory effect of *Portulaca* on QS is diketopiperazine. This compound has been reported as an inhibitor of the QS system in both *C. violaceum* CV026 and *P. aeruginosa* PAO1 [81]. It showed the inhibition of biofilm formation, a reduction in pyocyanin and elastase production in *P. aeruginosa* PAO1, and a reduced production of violacein in *C. violaceum* CV026 [82]. A third compound that might contribute to the inhibitory effect of *Portulaca* on the QS system is 6-hydroxy-4,4,7a-trimethyl-5,6,7,7a-tetrahydrobenzofuran-2(4H)-one (HTT). This compound belongs to a class of heterocyclic compounds known as furans, which possess a wide range of biological properties [54]. It has been reported that minor alterations in the substitution patterns of furan derivatives can lead to significant changes in their biological activity. This is evident in many agents with a core furan ring, representing antimicrobial compounds that also exhibit anti-inflammatory, analgesic, antidepressant, anxiolytic, anti-glaucoma, antihypertensive, diuretic, anti-ulcer, anti-aging, and anticancer properties [83]. The phytochemical analysis in this study also detected several phenols in the methanolic extract of *Portulaca*. The hydroxyl groups in the phenolic compounds were reported to disrupt transport across the bacterial cell membrane by altering the electron flow and inhibiting ATP synthesis, leading to cell death [84]. Some studies showed that specific polyphenols demonstrate anti-QS activity in *Pseudomonas putida* [85,86], while others indicated that phenolic compounds often demonstrate inhibitory effects on biofilm formation, swarming motility, and the production of some virulence factors, such as adhesion, proteolytic activity, and elastase [87,88,89].

This study showed a significant reduction in biofilm formation caused by *P. oleracea* methanolic extract. This inhibitory effect might be due to numerous extracted small phenols attenuating QS regulation in *P. aeruginosa*, as indicated by the large inhibitory zones formed and the lack of violacein produced by *C. violaceum.* Additionally, the phytochemical analysis showed the presence of several imidazolidine derivatives. Such compounds were reported as inhibitors of *P. aeruginosa* virulence factors, including protease, hemolysin, and pyocyanin [90]. This study reported that clinical isolates from patients with chronic infections may lead to the accumulation of mutations in QS genes, suggesting that a strain-specific response to QS inhibitors should be considered upon evaluating the anti-virulence properties of such compounds.

In conclusion, the findings of this study suggest the potential use of *P. oleracea* in managing infections caused by *P. aeruginosa* by inhibiting quorum sensing, and biofilm formation, and reducing the production of pyocyanin and LasA proteases. Natural compounds exhibiting quorum quenching may be combined with conventional antibiotics to enhance their efficacy as antimicrobials. To our knowledge, this study is the first to identify potential inhibitors of quorum sensing in *P. oleracea* which can suppress the growth of *P. aeruginosa* without causing stressful conditions. This study highlights *P. oleracea* as an important source of natural antimicrobials for possible use as a food additive, and in other pharmaceutical applications. Further investigation on the composition of additional active compounds in *P. oleracea* and their antimicrobial activity is recommended.

## Figures and Tables

**Table 1:** GC-MS spectral analysis of *Portulaca oleracea* methanolic extract.

Peak No.	*R*-Time (min)	Area (%)	Identification of Compound	Molecular Weight (g/mol)	Chemical Class	Formula	Activities	Reference
1	6.773	8.04	Benzene acetaldehyde	120.15	Aldehyde	C_8_H_8_O	Antioxidant, mutagenic, antimicrobial	[41]
2	7.844	4.81	2-pyrrolidinone	85.10	Lactam	C_4_H_7_NO	Induces cell line proliferation	[42]
3	8.220	5.17	Cyclopropane,2-chloro-1,1,3-trimethyl	118.60	Alkyl halide	C_6_H_11_Cl		
4	10.154	2.23	2-Methyl-1-octen-3-yne	122.2	Alkyne	C_9_H_14_		
5	10.684	3.59	2-Methoxy-4-vinylphenol	115	Phenol	C_9_H_10_O_2_	Antimicrobial, antioxidant, anti-inflammatory, analgesic, antigermination, antiproliferative	[43,44]
6	11.600	1.51	Trans-beta-Damascenone	190.28	Ketone	C_13_H_18_O	Anti-inflammatory, anticancer antispasmodic activity	[45,46]
7	12.87	1.07	4-(2,6,6-Trimethylcyclohexa-1,3-dienyl) but-3-en-2-one)	190.28	Ketone	C_13_H_18_O	Antioxidant	[44]
8	13.533	31.36	Sacrosine, N-cyclopropylcarbonyl-, propyl ester	199	Ester	C_10_H_17_NO_3_	Reduces cell viability	[47]
9	13.955	2.5	2,6-Dimethoxy-4-ethyl-phenol	180	Phenol	C_10_H_12_O_3_	Antioxidant capacity	
10	15.171	8.89	2,4-Imidazolidinedione,5-(2-methlypropyl)	156	Imidazolidine	C_7_H_12_N_2_O_2_	Antimicrobial, anticonvulsant	[48,49]
11	15.640	3.38	N-acetyl-3-phenylalanine methyl ester	221	Ester	C_12_H_15_NO_3_	Antimicrobial	[50]
12	15.783	1.81	Cyclo(L-prolyl-L-valine)	196	Diketopiperazine	C_10_H_16_N_2_O_2_	Antiproliferative activity	[51]
13	16.292	1.63	1-Phenyethyl-5-pyrrolidin-l-ylmethyl-1H-tetrazole	257	Tetrazole	C_14_H_19_N_5_	Anti-inflammatory, antidiabetic, anticancer, antibacterial activity	[52]
14	16.487	1.62	1,4-diazabicyclo [4.3.0]nonan-2,5-dione,3-methyl	168	Pyrimidine	C_8_H_12_N_2_O_2_	Antifungal, antimicrobial activity	[53]
15	16.648	1.56	6-hydroxy-4,4,7a-trimethyl-5,6,7,7a-tetrahydrobenzofuran-2(4H)-one (HTT)	196	Furan	C_11_H_16_O_3_	Anti-inflammatory	[54]
16	17.339	1.81	Cyclo(L-prolyl-L-valine)	196	Diketopiperazine	C_10_H_16_N_2_O_2_	Antiproliferative activity	[51]
17	18.117	3.20	2-Acetamido-3-phenylpropionamide	206	Phenylpropanamide	C_16_H_14_N_2_O_2_		
18	18.228	7.00	Benzyl alcohol, 3-benzyloxy-2-flouro	232	Alcohol	C_14_H_13_FO_2_	Antibacterial	[51]
19	18.358	1.56	Pyrrolo [1,2-a] pyrazine-1,4-dione, hexahydro-3-(2-methylpropyl)	210	Diketopiperazine	C_11_H_18_N_2_O_2_	Antioxidant, antifungal	[55]
20	18.526	1.2	5,10-Diethoxy-2,3,7,8 tetrahydro-1H,6H-dipyrrolo [1,2-a:1′,2′-d] pyrazine	250	Pyrazine	C_14_H_22_N_2_O_2_	Antimicrobial	[56]
21	20.751	0.64	Nonanamide, N-(1-benzyl, 2-phenylethyl)	351	Nonanamide	C_24_H_33_NO	Antimicrobial	[57]
22	22.134	2.11	Pyrrolo [1,2-a] pyrazine-1,4-dione, hexahydro-3-(2-methylpropyl)	244	Pyrazine	C_14_H_16_N_2_O_2_	Antioxidant, antibacterial	[58,59]

## Data Availability

The original contributions presented in this study are included in the article. Further inquiries can be directed to the corresponding author.
